# Direct Sensing of 5-Methylcytosine by Polymerase Chain Reaction**

**DOI:** 10.1002/anie.201403745

**Published:** 2014-06-12

**Authors:** Joos Aschenbrenner, Matthias Drum, Hüsnü Topal, Markus Wieland, Andreas Marx

**Affiliations:** Department of Chemistry, Konstanz Research School Chemical Biology, University of KonstanzUniversitätsstrasse 10, 78457 Konstanz (Germany)

**Keywords:** DNA methylation, DNA polymerases, enzyme engineering, polymerase chain reaction

## Abstract

The epigenetic control of genes by the methylation of cytosine resulting in 5-methylcytosine (5mC) has fundamental implications for human development and disease. Analysis of alterations in DNA methylation patterns is an emerging tool for cancer diagnostics and prognostics. Here we report that two thermostable DNA polymerases, namely the DNA polymerase KlenTaq derived from Thermus aquaticus and the KOD DNA polymerase from Thermococcus kodakaraensis, are able to extend 3′-mismatched primer strands more efficiently from 5 mC than from unmethylated C. This feature was advanced by generating a DNA polymerase mutant with further improved 5mC/C discrimination properties and its successful application in a novel methylation-specific PCR approach directly from untreated human genomic DNA.

Methylation of cytosines is a major epigenetic mark and the most abundant DNA modification in vertebrates.[1] Methylated cytosines are found as symmetrical 5-methylcytosine (5mC) of the dinucleotide CpG within promoter regions,[2] in which 75 % are methylated throughout the mammalian genome. In recent years, it has become evident that promoter methylation is crucial for activating and silencing gene expression.[3] Moreover, dynamic changes of methylation patterns are important for the development of mammals.[4] They control, for example, X-inactivation,[5] genomic imprinting,[5] and the development of primordial germ cells.[6] Furthermore, alterations in DNA methylation can be an integral event in the onset of diseases like cancer.[7] The discovery that particular hypo- or hypermethylation events are unique for human malignancy[8] renders 5mC a promising biomarker for cancer diagnosis. In reality, many DNA-methylation-based biomarkers have been evaluated in cancer research.[9] Therefore, several methods for the detection of 5mC in the human genome are employed.[10] The main approaches rely on endonuclease digestion,[11] affinity enrichment,[12] and bisulfite conversion.[13] The most common method to obtain single-nucleotide resolution is based on treatment of the sample with bisulfite, resulting in the conversion of cytosine to uracil whereas 5mC remains unchanged;[14] this is followed by DNA sequencing or PCR amplification. All available methods, however, have significant drawbacks. While affinity enrichment falls short in yielding information about individual CpG dinucleotides, endonuclease- and bisulfite-based methods are prone to false-positive results due to incomplete conversion.[15] The necessity of implementing pretreatment and purification steps results in a higher risk of contamination[16] as well as loss of DNA sample.[17] Taken together, the laborious, time-consuming,[18] and error-prone nature of DNA methylation assays is a significant barrier for their advent in practical clinical diagnostics. Here, we demonstrate the feasibility of a superior DNA methylation profiling approach directly from genomic samples.

In order to examine the behavior of two thermostable DNA polymerases when encountering a primer bearing a mismatch at its 3′-terminus opposite either C or 5mC, we investigated the extension from four primers differing in their 3′-terminal nucleotide (A-, C-, G-, or T-primer) paired with two different oligonucleotide templates that carry either a C or a 5mC opposite the primer end (Figure 1 b). Therefore, we performed single-nucleotide-incorporation experiments followed by analysis through denaturating polyacrylamide gel electrophoresis (PAGE) and visualization using autoradiography. Figure 1 c shows extensions of the four different primers by the sequence family A DNA polymerase KlenTaq.[19] Whereas there is no difference in extending from C or 5mC when the matched G-primer is used, the A- and T-primers are more efficiently extended in the case of the 5mC template. Under the chosen conditions, the extension for the A-primer was 56 % when paired with the 5mC template in comparison to 18 % when paired with the C template. The primer bearing C at its 3′-terminus was not extended for either template. Along these lines, we also tested an exonuclease-deficient variant of a DNA polymerase descending from another sequence family, the family B KOD DNA polymerase (KOD exo-)[20] originating from *Thermococcus kodakaraensis*. We found that discrimination between the mispaired C and 5mC templates is also observed with KOD exo-, albeit to a lower extent than with KlenTaq (47 % extension of the A-primer when paired with 5mC template as compared to 34 % with C template; Figure 1 d).

**Figure 1:**
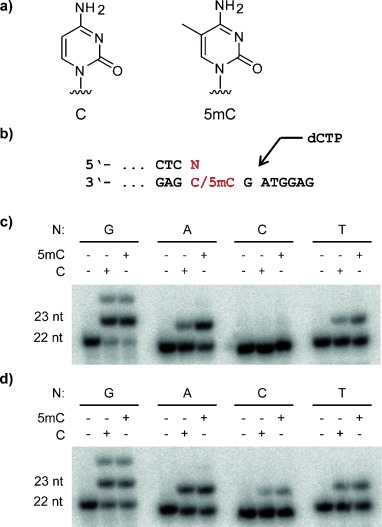
Comparison of KlenTaq and KOD exo- in single-nucleotide incorporation with different primers and either methylated or non-methylated template. a) Chemical structure of C (left) and 5mC (right). b) Partial primer template sequences used in primer extension experiments (N=G/A/T/C). c,d) Single-nucleotide incorporation catalyzed by KlenTaq (c) and KOD exo- (d) using primers bearing one of the four different nucleotides at the 3′-terminus opposite either C or 5mC. 100 μm dCTP and 25 nm of the respective DNA polymerase were applied. The reaction time was 60 s.

Next, we aimed at generating DNA polymerase mutants with enhanced discrimination. We inspected previously published KlenTaq and KOD exo- crystal structures with bound primer template complex,[21] looking for amino acids that might be able to interfere with the mismatched cytosine with the intention to exchange those by sterically more demanding residues. We came to the conclusion that the template binding cleft of KlenTaq is already packed with sterically demanding amino acids. For KOD exo-, however, we identified a glycine (G498) in immediate proximity to the mismatched cytosine (Figure 2 a). Noticeably, G498 is located in the template binding site where it is able to interact with the phosphate backbone of the nucleotide paired to the primer’s 3′-terminal nucleobase (Figure 2 a). We reasoned that mutating G498 into a sterically more demanding amino acid would result in a more crowded template binding site, which might effect discrimination between C and 5mC upon extension from mismatched primer strands. In fact, we found that mutating G498 into methionine results in a KOD exo- variant that exhibits the desired properties. The KOD exo- G498M mutant was obtained by site-directed mutagenesis followed by expression in *E. coli* BL21 as previously described.[21b, 22] The purified KOD exo- wild-type and KOD G498M enzymes were analyzed by SDS PAGE (Figure 2 b) and compared using the very same reaction conditions and enzyme concentrations. First, we applied KOD G498M in primer-extension experiments as described above. The engineered KOD G498M features considerably increased discrimination between the C and 5mC template as compared to that of the wild-type enzyme when the mismatched A-primer was used (44 % extension rate for 5mC as compared to 20 % for C) (Figure 3 a). This effect is even more pronounced for full-length primer extension. Here, 58 % of the applied primer was extended to the full-length product on the 5mC template as compared to 12 % on the C template (Figure 3 b). Interestingly, when the mismatched reaction was performed with template C a significant pausing of the enzyme was observed after incorporation of one nucleotide (Figure 3 b), a result that was not observed to this extent with the 5mC template. We determined steady-state kinetics[23] for primer extension by the incorporation of a single nucleotide (Table S1). All three enzymes were found to be equally efficient for either template when the matched G-primer was used (Table S1, Figure S1, and Figure S2). When comparing extension from the mismatched C template to the mismatched 5mC template for KOD exo- wild-type, we found that the discrimination is mainly based on differences of *K*_m_. In contrast, for KOD G498M as well as for KlenTaq wild-type we found that the differences are mainly based on *k*_cat_ (Table S1, Figure S1, and Figure 3 c,d).

**Figure 2:**
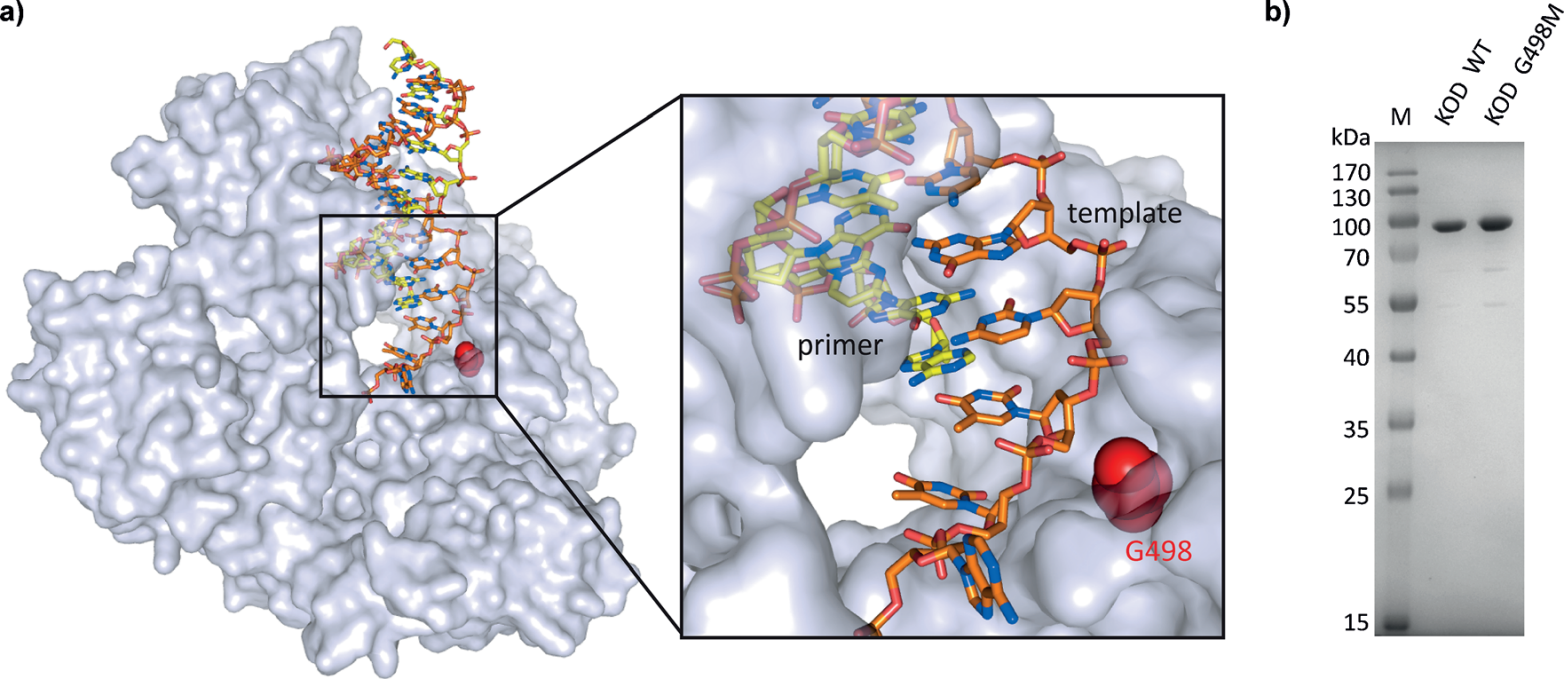
Rational design and preparation of a KOD mutant with enhanced 5mC discrimination. a) Crystal structure of KOD with bound primer template complex. Primer and template are shown as yellow and orange rods. G498 is highlighted as red spheres. Adapted from PDB 4K8Z[21b] using PyMOL (Schrödinger, LLC; New York, NY). b) Coomassie-stained SDS PAGE of Ni-NTA-purified 6×His-tagged KOD wild-type (middle) and KOD G498M (right).

**Figure 3:**
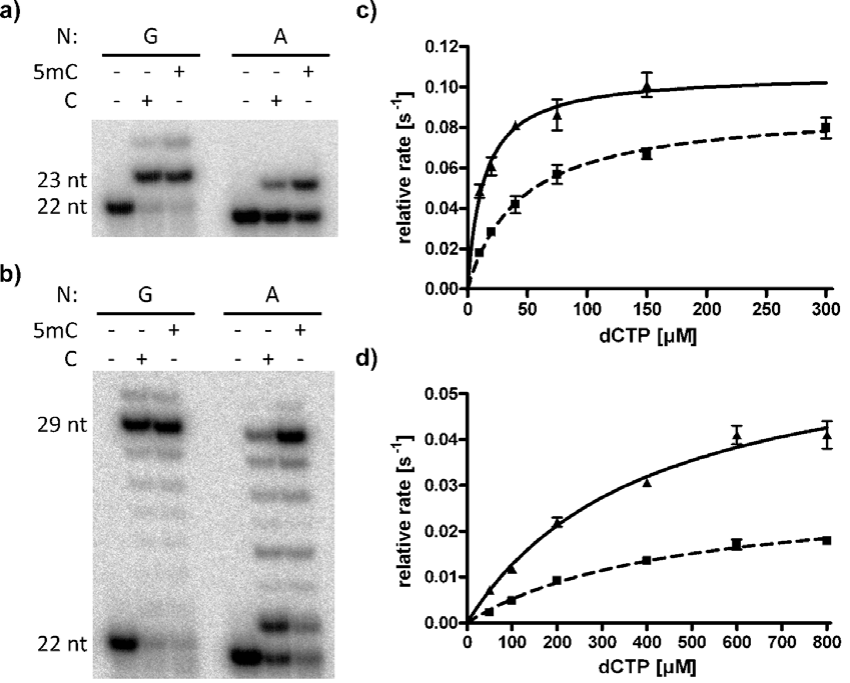
KOD G498M features enhanced 5 mC discrimination. a) Single-nucleotide incorporation with KOD G498M using primers bearing G or A at the 3′-terminus opposite either C or 5mC. 100 μm dCTP and 25 nm KOD G498M were applied. The reaction time was 5 min. b) Full-length primer extension experiment with KOD G498M using primers bearing G or A at the 3′-terminus opposite either C or 5mC. 100 μm dCTP and 25 nm KOD G498M were applied. The reaction time was 30 min. c,d) Steady-state kinetics of single-nucleotide incorporation next to A-mismatched C (dashed) and 5mC (solid) catalyzed by KOD exo- wild-type (c) and KOD G498M (d). Data points derive from triplicates. Error bars represent the standard deviation.

We examined the potential to exploit these differences in the catalytic efficiency of processing 5mC and C in PCR experiments on human genomic DNA (gDNA). Therefore, we analyzed a particular CpG site in the promoter region of NANOG in HeLa gDNA. NANOG is an epigenetically regulated gene which is associated with pluripotency of cells[24] and was found to be hypomethylated in its promoter region in metastatic human liver cancer cells.[25] Previous evaluation of the analyzed methylation site by bisulfite sequencing characterized it as unmethylated in HeLa cells.[25] As a control for full methylation we employed HeLa genomic DNA that was enzymatically methylated in vitro with a CpG methylase. We designed forward primers bearing either G or A at their 3′-termini opposite the cytosine of interest and a reverse primer binding 43 nt downstream so that PCR amplification should deliver a 86 bp amplificate (Figure S3). PCR employing the wild-type KOD exo- or KlenTaq enzymes did not show any differentiation in PCR efficiency. However, the use of KOD G498M DNA polymerase instead revealed that this variant is indeed capable of discriminating between methylated and unmethylated cytosine (Figure 4). While real-time PCR and melting curves are similar for untreated and CpG-methylated DNA when matched primers are used (i.e., terminating with G), employment of the mismatched primer (i.e., terminating with A) leads to delayed amplification as well as reduced endpoint fluorescence when the untreated DNA is compared to the CpG-methylated DNA (Figure 4 a). Melting curves of the amplified DNA confirm this decrease in PCR efficiency for the non-methylated template (Figure 4 b). Finally, quantitative analysis of the reaction mixtures by agarose gel electrophoresis substantiates these findings. As seen in Figure 4 c, the yield of a specific amplificate for the methylated template is reasonable considering we employed a mismatched primer. For the unmethylated template, however, the yield is significantly reduced. Moreover, KOD G498M is highly selective for the desired amplificate when the methylated template is used, whereas a slight band of byproduct appears when the unmethylated template is used. Primer dimers are formed in PCR without HeLa genomic DNA but do not emerge in the presence of template.

**Figure 4:**
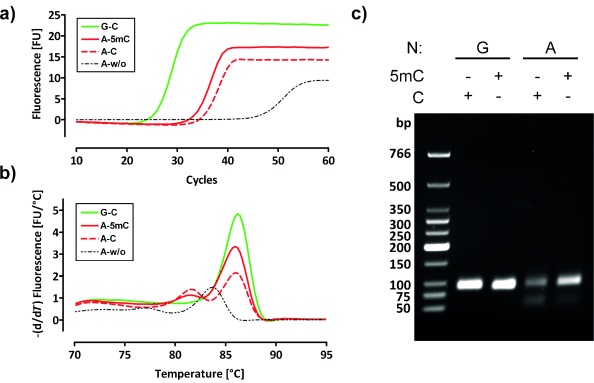
KOD G498M differentiates between methylated and non-methylated cytosine in PCR experiments from HeLa genomic DNA. a) Real-time PCR of the NANOG promoter region catalyzed by KOD G498M with matched (green) and mismatched primer (red) from HeLa gDNA (dashed) or CpG methylated HeLa gDNA (solid). b) Melting curves of amplificates deriving from (a). c) Agarose gel from PCR amplificates deriving from HeLa gDNA (C) or CpG methylated Hela gDNA (5mC) with matched (lane G) or mismatched primer (lane A). Curves and gels are representative for numerous experiments.

So far, detection of 5mC with single-base resolution has been restricted to the troublesome conversion of DNA by bisulfite or any other manipulation prior to analysis. Here we demonstrate the feasibility of a PCR system sensing 5mC directly from genomic DNA. The approach is based on the differential extension of mismatched primer strands by two well-studied DNA polymerases depending on whether the primer terminates opposite a template 5mC or C. KlenTaq was investigated as a prominent member of sequence family A and an exonuclease-deficient variant of KOD DNA polymerase as a representative of the sequence family B.[26] Both DNA polymerases are thermostable and commonly used in many core biotechnological applications. The discrimination between C and 5mC was greater with KlenTaq than with KOD exo-. Interestingly, KlenTaq wild-type exhibits a more sterically crowded environment close to the relevant template site in comparison to KOD exo-. This might well explain the enhanced discriminating properties of KlenTaq wild-type compared to KOD exo- wild-type. However, we were able to enhance the discrimination of KOD exo- by increasing the steric crowding of the template binding site in close vicinity to the residues bearing the C or 5mC of interest. Primer extensions in the presence of all four dNTPs indicate that KOD exo- G498M not only discriminates for C over 5mC upon extension by one nucleotide but also when the mismatch is already one position distal from the primer terminus (Figure 3 b). Furthermore, KOD G498M is capable of discriminating between C and 5mC in PCR from a genomic DNA target. On this basis, the methylation state of a single nucleotide in the entire human genome can be ascertained by a single PCR step.

As crystal structures of mismatched primer template complexes bound to DNA polymerases have not yet been described, one can only speculate about the origin of the 5mC/C discrimination. It has been proposed that when a mismatch is encountered, active misalignment of catalytic residues in the DNA polymerase results in reduced incorporation rate and therefore higher specificity of the enzyme.[27] Thus, discrimination could derive from differences in the gained substrate binding energy by misalignment of catalytic residues. Thereby, the enzyme would be kept in an inactive conformation more efficiently when bound to mismatched C rather than mismatched 5mC, resulting in a favored extension of 5mC. The differential states might be due to steric interference between the enzyme and the additional 5-methyl group in 5mC.

In the past, the possibility of different extension efficiencies was discussed as a mechanism for the increase of the mutation rates of 5mC in vivo. In 1992, Shen et al. investigated three DNA polymerases in their properties of extending mismatched primer strands at the C and 5mC position.[28] They found significant differences in mismatch extension only when using AMV reverse transcriptase. However, no significant discriminations were observed for a sequence family A DNA polymerase (i.e., the exonuclease-deficient Klenow fragment of *E. coli* DNA polymerase I) and a sequence family B enzyme (i.e., *Drosophila* DNA polymerase α). As mentioned, the herein investigated enzymes belong to sequence family A (KlenTaq) and B (KOD exo-) and show discrimination. These findings might hint at the fact that subtle changes of the enzyme scaffold might cause altered effects of C/5mC discrimination. Thus, future investigations will aim at evolving new DNA polymerase mutants with even more enhanced discrimination for application in methylation specific PCR approaches.[29]
