# Supplementary material for: Direct Sensing of 5-Methylcytosine by Polymerase Chain Reaction
Source: Angew Chem Int Ed Engl. 2014 Jun 12;53(31):8154–8. doi: 10.1002/anie.201403745 (PMC4499253; doi:10.1002/anie.201403745)
Supplement: Supplementary file 1 [file anie0053-8154-sd1.pdf]

Angewandte Chemie
Eine Zeitschrift der Gesellschaft Deutscher Chemiker

Supporting Information
© Wiley-VCH 2014
69451 Weinheim, Germany

Direct Sensing of 5-Methylcytosine by Polymerase Chain Reaction**

*Joos Aschenbrenner, Matthias Drum, Hüsnü Topal, Markus Wieland, and Andreas Marx**

anie_201403745_sm_miscellaneous_information.pdf

Supporting Information

Table of Contents:

Methods ... 1

Supplementary Data ... 3

Figure S1. Steady state Kinetic experiments with matched or A-mismatched primers and KlenTaq wild-type ... 3

Figure S2. Steady state Kinetic experiments with matched primers and KOD wild-type or KOD G498M ... 4

Figure S3. Primer design for PCR experiments on HeLa gDNA. ... 5

Table S1. Steady state kinetic analysis of nucleotide incorporation next to matched/mismatched C and 5mC by KlenTaq wild-type, KOD exo- wild-type and G498M. ... 6

References ... 7

Methods

Proteins, oligonucleotides and HeLa gDNA. Protein mutation was performed using site directed mutagenesis. Protein expression and purification was performed as described.[21, 22]. Purity of enzymes was validated by SDS-PAGE. Oligonucleotides were purchased from Biomers and Metabion and used directly for PCR experiments or purified by preparative denaturating PAGE. Radioactive labeling of primer strands was performed using [γ-^{32}P]-ATP and T4 polynucleotide kinase according to the vendors protocol. HeLa genomic DNA (gDNA) and CpG methylated HeLa gDNA was purchased from New England Biolabs and directly used for PCR.

Primer extension assay. The reaction mixture contained 150 nM of a [γ-^{32}P]-labeled primer (5'-d(GTT TCT CCA GTT TCT TTT CTC N)-3', N = A/C/G/T), 225 nM of either template (5'd-(GAG GTA GXG AGA AAA GAA ACT GGA GAA ACT CGG TG), X = C/5mC) and 25 nM of the respective DNA polymerase (KlenTaq, KOD exo- wild-type or KOD G498M) in buffer (50 mM Tris HCl pH 9.2, 16 mM $(NH_4)_2SO_4$, 1.75 mM $MgCl_2$, 0.1% Tween 20). Reaction mixtures (20 µl, respectively) were heated to 95°C for 2 min and subsequently cooled to 55°C. After starting the primer extension by addition of 100 µM dCTP for single nucleotide incorporation or 100 µM of each dNTP for full length primer extension, reactions were allowed to proceed at 55°C for the desired incubation time (see legends of Fig. 1 and 3). Reactions were stopped by addition of 40 µl stop solution (80% (v/v) formamide, 20 mM EDTA, 0.25% (w/v) bromophenol blue, 0.25% (w/v) xylene cyanol) and analyzed by 12% denaturating PAGE. Visualization was performed by phosphorimaging.

Enzyme kinetics. Steady state kinetics of KOD exo- wild-type and KOD G498M with matched and 3'-mismatched primers were measured under single completed hit conditions[23]. Single nucleotide incorporation for the respective primer in complex with either template (as above) was performed as described, analyzed via 12% denaturating PAGE and visualized by phosphorimaging. DNA polymerase concentrations were chosen so that not more than 20% of the applied primer was extended. The rate of single nucleotide incorporation was determined for various incubation times (10s – 60s) at various dCTP concentrations. The amount of extended primer was plotted against incubation time for each examined dCTP concentration. Linear regression delivered reaction velocities for the respective dCTP concentration. For kinetic analysis, the reaction velocities were normalized against enzyme concentrations and plotted against the employed dCTP concentrations. Using GraphPad Prism 4, experimental data was fit to a hyperbolic equation [velocity] = k_{cat}*[dCTP]/(K_M + [dCTP]) to determine K_M and k_{cat}:

PCR experiments. Reaction mixtures for PCR contained 200 µM dNTPs (each), 50 nM forward primer (5'-d(CTT GGT GAG ACT GGT AGA CN)-3', N = A/G), 100 nM reverse primer (5'-d(TTA GAC CCA CCC CTC CTG GCG)-3'), 150 nM KOD G498M, 1x sybr green I (Sigma) and 2 ng/µl of either HeLa gDNA or CpG methylated HeLa gDNA in buffer (50 mM Tris pH 8, 16 mM $(NH_4)_2SO_4$, 30 mM NaCl, 1.5 mM $MgCl_2$ and 0.1% Tween 20). Real time PCR data was gathered from respectively 25µl reaction mixture using the Roche LightCycler® 480 system with an initial denaturation at 95°C for 5 min followed by amplification over 60 cycles with denaturation at 95°C for 10s and annealing and elongation at 68°C for 30s. Melting curves were measured immediately after PCR amplification. Subsequently, reaction mixtures (10 µl respectively) were analyzed using agarose gel electrophoresis and visualization by EtBr staining and UV irradiation.

Supplementary Data

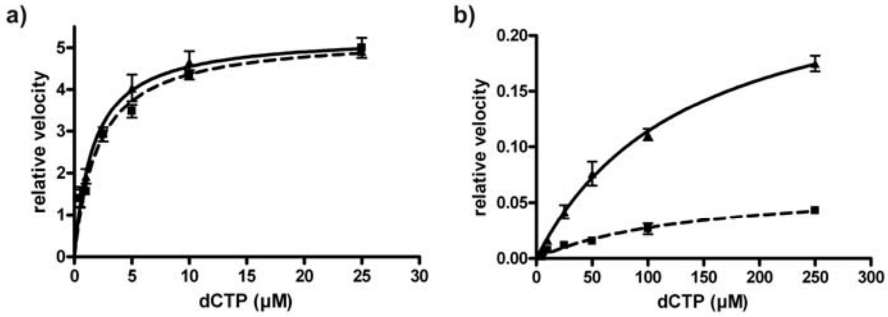

Figure S1. Steady state Kinetic experiments with matched or A-mismatched primers and KlenTaq wild-type. a,b) Steady state kinetics of single nucleotide incorporation next to matched (a) or A-mismatched (b) C (dashed) or 5mC (solid) catalyzed by KlenTaq.

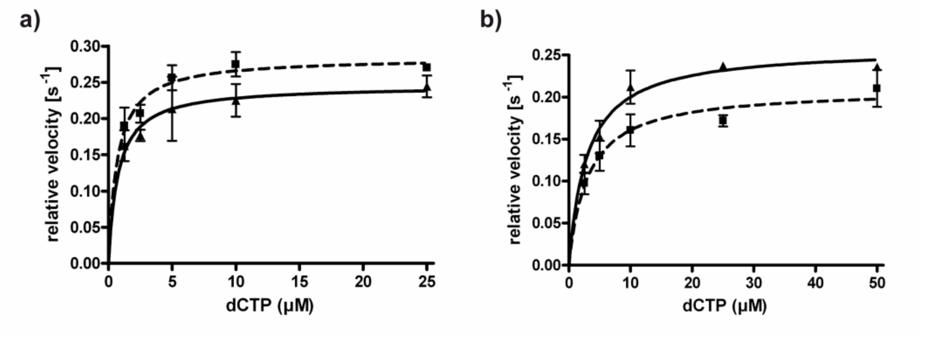

Figure S2. Steady state Kinetic experiments with matched primers and KOD wild-type or KOD G498M. a,b) Steady state kinetics of single nucleotide incorporation next to matched C (dashed) or 5mC (solid) catalyzed by KOD wildtype (a) and KOD G498M (b).

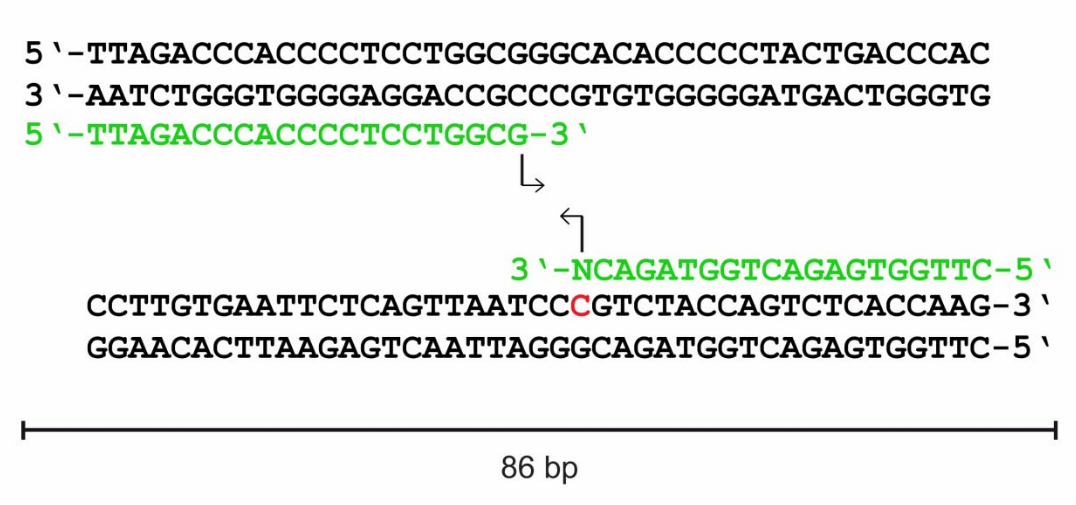

Figure S3. Primer design for PCR experiments on HeLa gDNA. Sequence of the amplified NANOG promoter region. The analyzed methylation site is highlighted in red. PCR primers are depicted in green (N = G/A)

Table S1. Steady state kinetic analysis of nucleotide incorporation next to matched/mismatched C and 5mC by KlenTaq wild-type, KOD exo- wild-type and G498M.

DNA polymerase	3‘ nucleotide of primer	template	k_{cat}[a] [s^{-1} x 10^{-2}]	K_M[a] [µM]
KlenTaq wild-type	G	C	528 ± 26	2.10 ± 0.37
KlenTaq wild-type	G	5mC	531 ± 16	1.69 ± 0.18
KlenTaq wild-type	A	C	6.63 ± 0.84	140 ±11
KlenTaq wild-type	A	5mC	26.9 ± 1.1	136 ± 35
KOD exo- wild-type	G	C	28.4 ± 1.0	0.692 ± 0.152
KOD exo- wild-type	G	5mC	24.5 ± 0.8	0.749 ± 0.144
KOD exo- wild-type	A	C	8.95 ± 0.21	44.3 ± 3.1
KOD exo- wild-type	A	5mC	10.7 ± 0.4	13.5 ± 2.2
KOD exo- G498M	G	C	21.0 ± 1.1	3.06 ± 0.50
KOD exo- G498M	G	5mC	25.9 ± 1.0	2.93 ± 0.67
KOD exo- G498M	A	C	2.91 ± 0.23	462 ± 75
KOD exo- G498M	A	5mC	6.39 ± 0.58	401 ± 80

[a] Data points derive from triplicates. ± describes SD.

References

[21] a) K. Betz, D. A. Malyshev, T. Lavergne, W. Welte, K. Diederichs, T. J. Dwyer, O. Phillip, F. E. Romesberg, A. Marx, *Nat. Chem. Biol.* **2012**, *8*, 612-614; b) K. Bergen, K. Betz, W. Welte, K. Diederichs, A. Marx, *ChemBioChem* **2013**, *14*, 1058-1062.

[22] a) S. Obeid, N. Blatter, R. Kranaster, A. Schnur, K. Diederichs, W. Welte, A. Marx, *EMBO J.* **2010**, *29*, 1738-1747; b) K. Betz, F. Streckenbach, A. Schnur, T. Exner, W. Welte, K. Diederichs, A. Marx, *Angew. Chem. Int. Ed.* **2010**, *49*, 5181-5184.

[23] a) M. S. Boosalis, J. Petruska, M. F. Goodman, *J. Biol. Chem.* **1987**, *262*, 14689-14696; b) S. Creighton, M. M. Huang, H. Cai, N. Arnheim, M. F. Goodman, *J. Biol. Chem.* **1992**, *267*, 2633-2639; c) J. Petruska, M. F. Goodman, M. S. Boosalis, L. C. Sowers, C. Cheong, I. Tinoco, *Proc. Natl. Acad. Sci. USA* **1988**, *85*, 6252-6256.
